# Mechanistic Explanations for Restricted Evolutionary Paths That Emerge from Gene Regulatory Networks

**DOI:** 10.1371/journal.pone.0061178

**Published:** 2013-04-17

**Authors:** James Cotterell, James Sharpe

**Affiliations:** 1 EMBL-CRG Systems Biology Research Unit, Centre for Genomic Regulation (CRG), Barcelona, Spain; 2 Developmental Biology Section, MRC Human Genetics Unit, Edinburgh, United Kingdom; 3 Institució Catalana de Recerca i Estudis Avançats (ICREA) Professor, Centre for Genomic Regulation (CRG), Barcelona, Spain; University of Iceland, Iceland

## Abstract

The extent and the nature of the constraints to evolutionary trajectories are central issues in biology. Constraints can be the result of systems dynamics causing a non-linear mapping between genotype and phenotype. How prevalent are these developmental constraints and what is their mechanistic basis? Although this has been extensively explored at the level of epistatic interactions between nucleotides within a gene, or amino acids within a protein, selection acts at the level of the whole organism, and therefore epistasis between disparate genes in the genome is expected due to their functional interactions within gene regulatory networks (GRNs) which are responsible for many aspects of organismal phenotype. Here we explore epistasis within GRNs capable of performing a common developmental function – converting a continuous morphogen input into discrete spatial domains. By exploring the full complement of GRN wiring designs that are able to perform this function, we analyzed all possible mutational routes between functional GRNs. Through this study we demonstrate that mechanistic constraints are common for GRNs that perform even a simple function. We demonstrate a common mechanistic cause for such a constraint involving complementation between counter-balanced gene-gene interactions. Furthermore we show how such constraints can be bypassed by means of “permissive” mutations that buffer changes in a direct route between two GRN topologies that would normally be unviable. We show that such bypasses are common and thus we suggest that unlike what was observed in protein sequence-function relationships, the “tape of life” is less reproducible when one considers higher levels of biological organization.

## Introduction

It remains unclear how restricted evolving populations are to move through any route in genotype space. This is due to our lack of understanding of how genotype maps to phenotype, since mutations occur at the level of genotype yet selection acts at the level of phenotype. In particular it has been suggested that certain combinations of genetic interaction are not viable or less fit, meaning that evolution cannot tinker through any form; there are restrictions on trajectories. Evolutionary trajectories can be viewed through the guise of a neutral network which derives from Maynard-Smith’s original concept of protein space [1]. A neutral network assumes that genotypes fall into two classes; those that are viable and those that are non-viable. Only those that are viable are included in the neutral network, and structurally similar genotypes are connected based on particular criteria. The shape of a neutral network can lie between two extremes: from regular and smooth to irregular with concavities. Only on irregular neutral networks are evolutionary trajectories restricted since the path to another viable genotype may involve a step to a non-viable genotype. Indeed neutral networks were first utilized to explore evolutionary routes in RNA sequence-shape space [2]–[4].

**Figure 1:**
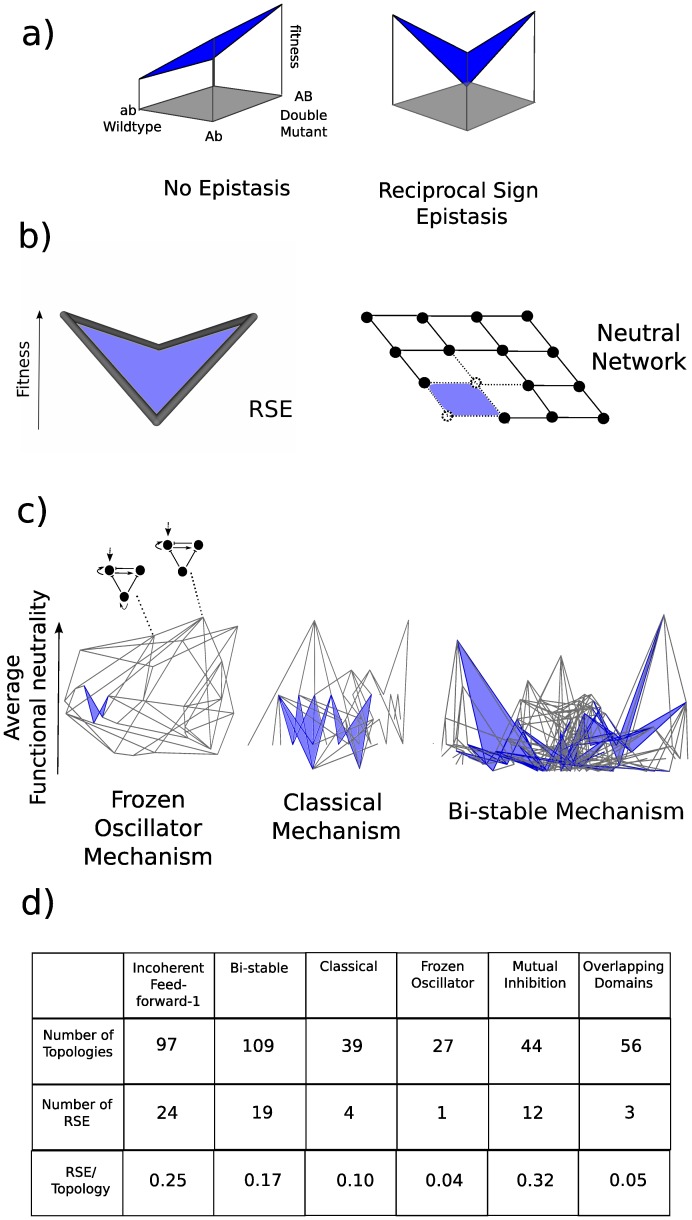
Epistasis and neutral networks. a) Illustrating reciprocal sign epistasis. When there is no epistasis the combined effects of two mutations are the result of the addition of the fitness effect of each individual mutation. There is reciprocal sign epistasis when the two individual mutations negatively affect fitness yet the double mutant is fitter than the combination of individual mutations. Figure adapted from [15]) b) Illustrating how reciprocal sign epistasis causes the irregularity in the shape of a neutral network. Dots are viable genotypes and edges connect genotypes equal except for one mutation. We assume the two unfit genotypes in an RSE geometry are unviable (dashed dots) and the two fit genotypes are viable. Therefore RSE will be responsible for the gaps in neutral networks that lead to the irregular shape (loss of the dashed edges and dots in the neutral network shown). c) Examples of average functional neutrality landscapes for 3 gene regulatory network mechanisms capable of interpreting a morphogen gradient. Topologies are vertices and single mutant neighbors are connected via edges (as illustrated by the two topologies in the Frozen Oscillator average functional neutrality landscape). Topologies are spaced in the y-axis according to their average functional neutrality (fraction of viable parameter space capable of performing the morphogen interpretation function) and in the x-axis to reduce edge-crossing. Mutant neighborhoods where statistically significant RSE exists are colored blue. d) The number of topologies and the incidence of RSE for each of the different mechanisms. The amount of RSE normalized to the number of topologies in the landscape can be found on the bottom row.

Irregular neutral networks are caused by the phenomenon of epistasis (Figure 1a and b). Epistasis essentially means that the fitness of certain genetic combinations does not have a simple linear relationship to the component parts [5]–[8]. A particular form of epistasis, *reciprocal sign epistasis* (RSE), describes a 2-loci situation where a wildtype and a double mutant are viable or fit yet the corresponding single mutants are unviable or significantly less fit. In particular this type of epistasis captures the basic idea of restricted evolutionary paths since no direct route is possible between the two viable/fit genotypes. The study of reciprocal sign epistasis is thus important to understand how evolution is constrained. Important questions include: How prevalent is RSE in nature? What is the underlying mechanistic basis? And can it be bypassed?

Previous studies exploring how epistasis affects evolutionary trajectories have mainly focused on sequence-function relationships in enzymes [9]–[16]. However since selection generally acts at the level of whole organisms, and multicellular phenotypes are largely controlled by gene regulatory networks (GRNs) it therefore remains an important challenge to go beyond interactions within single genes/proteins, and to consider possible epistasis within the context of dynamical GRNs. We therefore chose a biologically-validated model of how GRNs can create multicellular spatial patterns as a new paradigm within which to explore the extent and the mechanistic basis of RSE in developmental processes. We previously employed such a biologically-validated model of gene regulation to explore how cells can arrange themselves into organized domains of expression state, by interpreting a morphogen gradient to generate a stripe of gene expression (File S1 and Figure S1) [17]. We chose the most basic functional measure; simply whether the GRN (genotype) produces a stripe of gene expression or not giving a simple binary fitness score of 0 or one for each genotype which defines whether it is included in the neutral network or not. Because of the continuous model of gene regulation used here, the resulting stripes of gene expression can have different sizes and shapes (and still be considered functional; See Methods). As such the functional genotypes are “nearly” neutral though we will refer to them as neutral throughout this manuscript for purposes of clarity.

Gene regulatory network (genotype) space is not naturally discrete like protein or RNA space. Protein or RNAs have discrete amino acids or bases at each sequence position unlike gene regulatory networks that have a continuous parameter range at each gene-gene interaction position. One way to make genotype space discrete is to use a representation known as topology space, which is based on the gene network structure or wiring design (See File S1 and Figure S2). A Step within topology space then is a discrete addition/removal of a single gene-gene interaction. All genotypes with a given topology, regardless of their underlying parameter values map to that point in the corresponding topology space. We devised a “topology atlas” – a metagraph (a graph of graphs) which represents all possible topologies, and directly links together those which have just a single topological difference. Such metagraphs have been shown to be useful for exploring the relationship between innovation and robustness in GRNs [18], [19].

A topology represents a population of individual GRNs with a particular wiring structure. If one samples a number of random parameter sets for each topology (each parameter set is an individual genotype) and sums the resulting binary fitness scores then an average functional neutrality score is generated for that region of genotype space (which is also a measure of robustness with respect to parameter sets for the corresponding topology). These average functional neutrality landscapes are thus a form of abstract neutral network that contain information about the number of functional individuals within that region of genotype space. We postulated that this average functional neutrality should correlate with the number of functional paths through that region of genotype space represented by the corresponding topology. Hence we used the average functional neutrality landscape of discrete topologies as a tool to measure the frequency of RSE and restricted evolutionary paths within the underlying neutral network of continuous genotypes.

## Results

To measure the prevalence of RSE in this system, we first simulated every topology from the topology atlas with 30,000 random parameter sets. Functional topologies are those that could produce a stripe of gene expression for at least one tested parameter set (See Methods for the definition of functionality). In the previous study we found that the vast majority of topologies were working by one of 6 different dynamical mechanisms. We split the functional topologies into the different mechanism groups and analyzed each group separately (See Methods and Supplementary Methods). This was required since the different mechanisms are using distinctly different regions of parameter space [17]. As such it is not appropriate to analyze RSE with all topologies included in one set since it is probably not possible to mutate between genotypes of differing mechanism and maintain function at the level of genotype (topology with a specific parameter set). In other words the different mechanisms probably represent disconnected neutral networks at the level of genotype and thus it is appropriate to analyze them separately.

We then defined the average functional neutrality of a topology as the fraction of parameter space that is functional (that can produce a stripe of gene expression). We systematically searched through the list of successful GRNs for pairs (reference topologies) that are 2 steps apart in the atlas, but whose 2 direct intermediate topologies both display significantly lower average functional neutrality (i.e. we searched for groups of 4 connected topologies whose surface in the average functional neutrality landscape displays the V-shaped fitness geometry typical of RSE in Figure 1a). Statistically significant lower average functional neutrality was defined as a threshold of one standard deviation of the average functional neutrality of the reference topologies assuming a bootstrapped binomial distribution (see Methods). This analysis revealed that RSE is widely-distributed across average functional neutrality landscapes for all 6 mechanisms (see Figure 1c for 3 examples) however its prevalence appears to vary between the different mechanisms (Figure 1d). This variation is not simply the result of different sized landscape – indeed the proportion of topologies involved in RSE changes between the different mechanisms. This suggests that to achieve the same biological function a choice exists between different dynamical mechanisms, which may possess average functional neutrality landscapes with intrinsically smoother or more irregular distributions.

**Figure 2:**
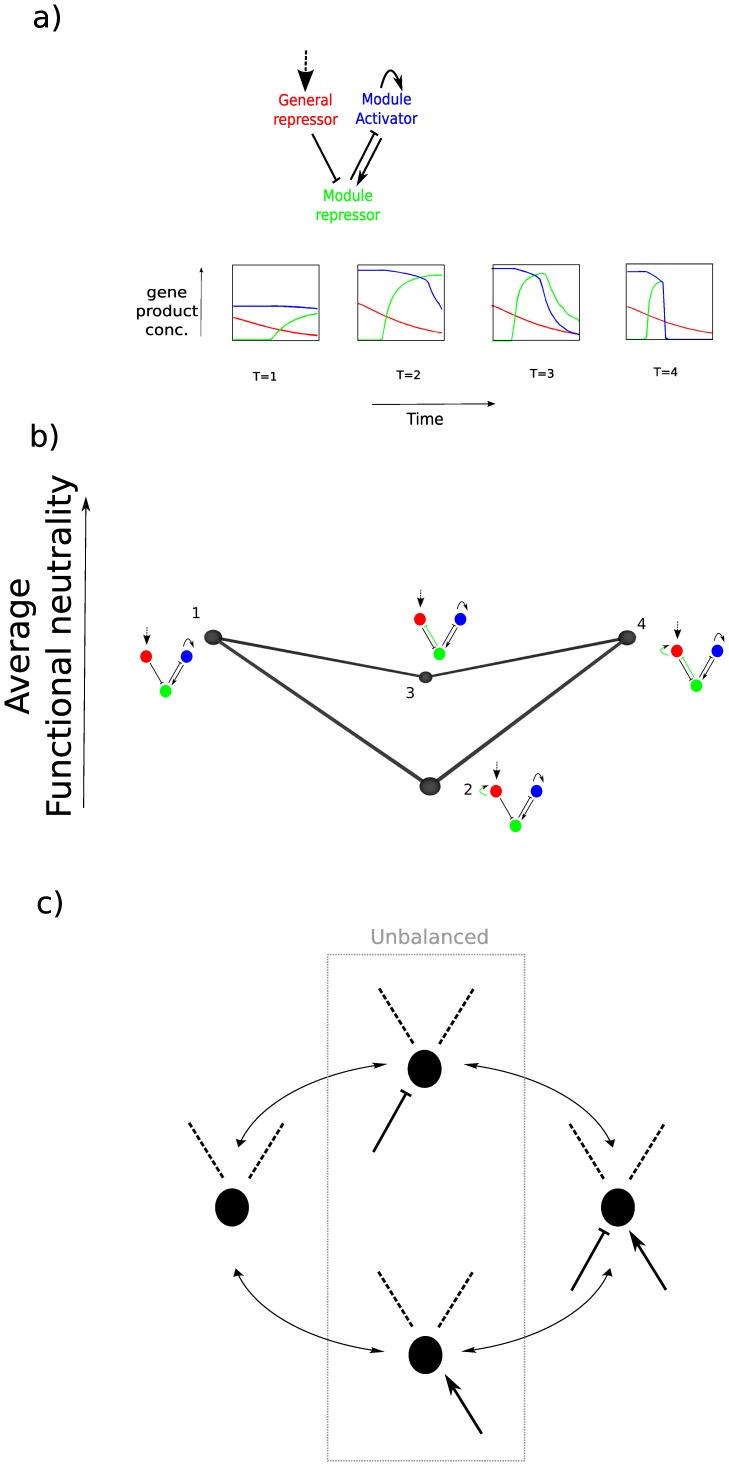
Counter-balanced gene regulatory inputs as a cause of RSE. a) A description of the bi-stable mechanism. (Top) The core topology. The genes are named based on their role in the mechanism. The mechanism involves a “general repressor” which regulates the activity of a bi-stable module. The bi-stable module consists of an auto-activating gene that activates a gene that represses itself. (Bottom) The space time behavior of the core topology with a typical parameter set. The x-dimension represents space and the y-dimension represents the gene product concentration. The color corresponds to the gene in the topology above. T indicates the representative stage of the mechanism. The morphogen feeds into the general repressor that correspondingly forms a similar gradient. The “module activator” starts to be expressed everywhere due to positive auto-regulation. The “module repressor” starts to be expressed on the right hand side due to activation everywhere by the module activator and repression on the left hand side by the general repressor. *Middle (T = 2):* At the very right hand side, the module repressor reaches a high enough concentration to start to force the activator off. *Late (T = 3):* The module repressor concentration also starts to drop due to lack of activation from the module activator. *Final (T = 4).* The result is a stripe of expression of the module repressor gene. b) An example of where counter-balanced gene-gene regulatory inputs cause RSE. The core topology of the Bi-stable mechanism (black gene-gene interactions) is viable/fit (1) along with the core topology with two additional interactions of opposing sign (green gene-gene interactions) that feed into the same gene (4). However either of the individual mutations alone has significantly less average functional neutrality (2 and 3). c) An illustration of the general concept of counter-balanced gene regulatory inputs causing RSE. The inputs must feed into the same gene though the interaction could come from the same gene or another gene.

What then is the mechanistic cause of RSE within our spatial patterning GRNs? To address this question, we studied in detail a local region of the average functional neutrality landscape responsible for the Bi-stable mechanism (Figure 2a). This local region displays the V-shaped viability/fitness valley due to RSE – the direct routes between two functional GRN topologies show extremely low average functional neutrality (Figure 2b). All versions of this mechanism display a common underlying core topology (black regulatory interactions in Figure 2b). One of the two viable versions of the topology (topology 1) contains only this core design, while the other viable version (topology 4) contains two extra regulatory links (green arrows). Importantly these two extra regulatory links are counter-balanced; that is they are opposite in sign and affect the same gene. By analyzing the pattern-forming dynamics of the networks, we discovered that these counter-balanced regulatory inputs explain the observed RSE. With only the activating input (topology 2), or only the repressive input (topology 3), the mechanism is unbalanced and is unable to correctly interpret the morphogen gradient (Figure 2c). However, when both regulatory inputs are present, the system is balanced, and able to form a stable stripe of expression.

Are counter-balanced regulatory inputs a general cause of RSE in dynamical networks, or is our example a specific one-off? To address this question, we explored how many of the RSE examples found for our 6 mechanisms were explained in the same way. For each case of RSE described earlier, we analyzed what mutational changes there are in the two intermediates. If one of those mutational changes is the addition of a positive interaction feeding into a particular gene, and the other is a repression feeding into the same gene, then we classified this as an example of counter-balanced regulatory inputs. We find that counter-balanced regulatory inputs underlie RSE in most of the 6 mechanisms studied. Interestingly however, it is mechanism dependent, for example it was never observed for the Classical mechanism, but explains 36% of RSE in the Bi-stable average functional neutrality landscape (when the topologies of the Bi-stable average functional neutrality landscape are re-sampled with a million parameter sets). We have thus uncovered a likely common cause of RSE underlying biological systems, but which is influenced by the specific dynamical design of the GRN.

**Figure 3:**
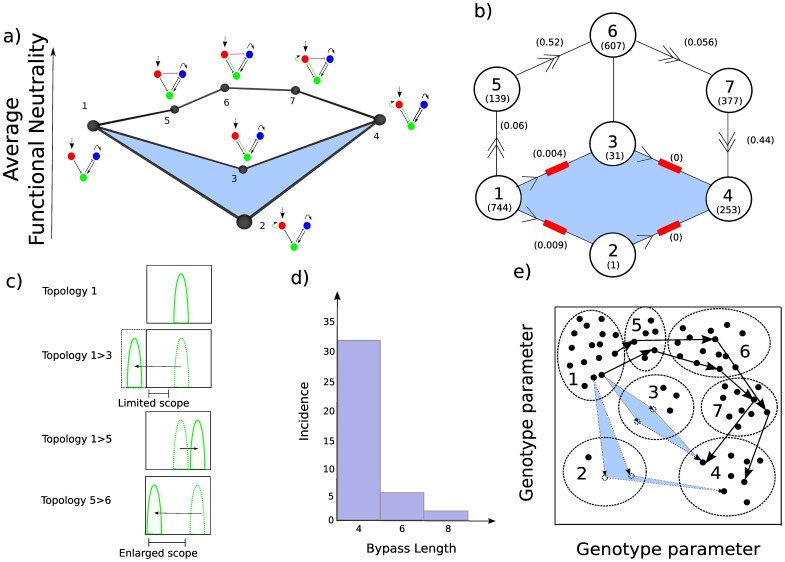
Permissive mutations allow RSE to be bypassed. a) The same example of RSE from figure 2 with an extra-dimensional bypass. The addition of a permissive mutation (red interaction in topology 5) before the original individual mutations are added (green interactions in topologies 6 and 7) allows the topology to retain a high average functional neutrality. The permissive mutation can then be removed (7 to 4) resulting in a bypass of the RSE. b) A scheme adapted from Ortlund et al., (2007) highlighting the similarity of permissive mutations in gene regulatory networks and in enzyme sequence-function relationships [14]. Topologies are shown in the circles. Large numbers in the circles represent the topology number and the number in the bracket is the number of functional parameter sets for that topology (i.e. the average functional neutrality; note that this was measured after the 7 topologies were re-simulated with a million parameter sets). The numbers on the edges represent the probability of a mutation of one of the successful parameter sets being functional when changed to the next topology when stepping through parameter space (in the direction of the arrows; see Methods). Non-viable steps are shown as red blocks (defined by a chance of less that 1%) and viable steps are shown by double headed arrows. c) The permissive interaction is caused by opposing effectors of differing strength. A typical mutation from a genotype with topology 1 to a genotype with topology 3 will significantly shift the stripe of gene expression to the left (second panel). The majority of these mutations leave the stripe outside the spatial field as defined by the morphogen gradient and therefore they are not functional. The mutation of a genotype with topology 1 to a genotype with topology 5 on the other hand typically shifts the stripe to the right and by a much smaller amount than the 1>3 shift (see main text). Therefore many more of the 1>5 transitions are viable. Because many more of the genotypes that correspond to topology 5 have their stripes at the extreme of the spatial domain, there is now more scope to introduce the green inhibition of topology 3. There is more space to shift the stripe by introducing this interaction and still result in a functional stripe. d) A histogram of the extra-dimensional bypass lengths between pairs of topologies in RSE. e) An illustration of how an average functional neutrality landscape can be envisaged as a density map of functionally neutral genotypes and how our measure of RSE and bypasses using topology is an underestimate. The Axes are genotype parameters. In reality there are 3 dimensions for the 3 interactions added between topology 1 and 7 but here we have reduced to 2 for simplicity. Black dots represent functional genotypes (topology with specific parameter set) and dashed dots represent non-functional genotypes. Large dashed circles represent the topologies (numbers) to which those genotypes map. Multiple different RSE and extra dimensional bypasses between topology 1 and 4 are shown by the light blue RSE geometry and the paths of solid black arrows respectively.

The widespread appearance of RSE suggests that the underlying neutral networks at the level of continuous genotype space are irregular and that evolutionary paths will be restricted. Irregular neutral networks can be considered an abstract version of rugged fitness landscapes. However it has been suggested in that the appearance of rugged fitness landscapes is simply the result of not taking into account all of the appropriate genetic loci that describe a particular biological system [20]. When the appropriate number of genetic loci are taken into account so that there are more than 3 dimensions required to describe a fitness landscape, fitness ridges exist between peaks in the higher dimensions. Gavrilets (2004) has described those paths through unaccounted for dimensions that connect fitness peaks as “extra-dimensional bypasses”. Such bypasses have been shown to exist in the contexts of inflorescence architectures, flower color and enzyme sequence-function relationships [14], [21], [22]. Ortlund *et al.,* 2007 in the context of enzyme sequence-function relationships have demonstrated how a “permissive” mutation allowed an unfavorable mutation to occur without loss of function. The presence or absence of the permissive interaction can be thought of as an extra dimension in genotype space [16], [20]. Returning to our example of RSE from the Bi-stable mechanism, we too see that a permissive mutation is possible: Adding a repressive interaction to the network (red link in Figure 3a) “buffers” the system, allowing one of the mutations that previously resulted in a low average functional neutrality to be incorporated (green repressive link). From this design, the second of the epistatic mutations is also viable, and from the resulting topology (7) the buffering link can be removed. Hence higher-dimensional fitness ridges exist in both enzyme sequence-function relationships and GRNs responsible for pattern formation. The underlying similarity of the landscape structure can be seen by comparing Figures 3a and 3b.

We next investigated exactly how the permissive interaction maintains the functionality of topology 1 when the direct interactions are added. The key to understand how the permissive mutation allows for a bypass is to consider how the stripe of gene expression moves in the spatial domain when the two inhibitory mutations are added from topology 1 to topology 3 and 5. To test the affect on stripe position of the topology 1>3 and 1>5 transitions we mutated all functional parameter sets of topology 1 into topology 3 and 5 by giving them the appropriate interaction. We gave all parameter sets a small strength interaction (0.1) so that we could see the typical movement of the position of the stripe without losing function. We simulated the un-mutated and mutated parameter sets and asked how much the peak (highest value) of the stripe moves. The results are striking and show that on average mutating to topology 5 moves the peak 1.1 cells to the right, whereas mutating to topology 3 on average moves the peak 8.3 cells to the left for an inhibitory interaction of 0.1. This means that typically a mutation to topology 3 will destroy the function since most mutations involve a change far greater than 0.1 which will shift the stripe out of the spatial configuration of the model (off the left hand boundary as depicted in Figure 3c second panel). However a mutation to topology 5 will shift the stripe several cells to the right and most probably maintain functionality (Figure 3c third panel). That means that typically parameter sets from topology 5 will have their stripes further to the right of the spatial domain. Such a configuration gives much more scope for functionality when one adds the inhibition of topology 3 to generate topology 6 as depicted in Figure 3c (bottom panel). There is now more space to shift the stripe of expression and maintain functionality. Hence the permissive mutation functions by having a small counteracting affect to the detrimental mutation thus improving the likelihood that the system will stay within the spatial configuration of the model when the permissive mutation occurs before the detrimental mutation.

The observation of higher dimensional fitness ridges in multiple contexts suggests that the concept of 2-loci epistasis may not be appropriate for the study of many biological systems. This point depends on whether these extra-dimensional bypasses are a common feature of the neutral networks that underlie biological systems? To explore how common extra dimensional bypasses are in these situations where there is RSE, we analyzed the extent of bypasses between the RSE topologies of the Bi-stable mechanism. A bypass must only contain topologies with significant average functional neutrality (using the same significance measure described earlier) and can be of any length. The results are shown as a histogram in Figure 3d. In 71% (40 out of 56) of the cases of RSE a high fitness ridge exists. Hence extra dimensional bypasses are almost as common as RSE since in most cases where we observe RSE there is also a route between the two topologies that does not involve a significant average functional neutrality decrease. Those situations of RSE where there is a bypass of length 4 have a direct permissive mutation between them. In other words like the example in Figure 3a, the two direct routes result in loss of fitness but the addition and removal of a single gene-gene interaction buffers these changes allowing a transition without loss of average functional neutrality. The longer (>4) bypasses between the two RSE topologies involve more complex buffering than simple single permissive mutations such as multiple subtle effects of individual mutations.

As mentioned in the introduction we postulated that the average functional neutrality of a topology should correlate with the number of functional paths through that underlying region of genotype space. To directly test this postulation we attempted to mutate each functional genotype (topology with specific parameter set) belonging to one topology class directly into the neighboring functional topology class by the addition or removal of a single gene-gene interaction with the rest of the parameter set equal. For each functional parameter set of each topology we added/removed the appropriate interaction which was given a random strength (See Methods). The resulting parameter set was simulated after each mutation and the stripe criterion was used to ask whether the mutant genotype was functional. We performed 100 mutations for each functional genotype and scored the fraction of mutations that were functional for each topology (labeled on the edges of Figure 3b). The result demonstrates that it is far more probable (approximately 10 fold comparing transition 1>5 versus 1>2 or 1>3) to generate successful mutants moving through the bypass than through the direct routes. This result shows that there is a larger fraction of functional paths through the topologies that have larger average functional neutrality. The probability that a mutation is functional and the fraction of functional parameter space of the resulting topology have a Pearson’s correlation coefficient of 0.74 (one-tailed probability of 0.046). Furthermore we can perform walks in parameter space that change topology 1 to 4 all the way through the bypass that maintain functionality yet none of an equal number of walks through the direct routes maintain functionality (See File S1 and Figure S3). Together these results validate our postulation and the use of topology space as a meaningful way of measuring RSE and restricted evolutionary paths in GRNs.

## Discussion

In summary, we have taken the analysis of RSE into the realm of dynamical GRNs. We demonstrate that most mechanisms for performing even a simple developmental function display some degree of RSE in their underlying functionality landscapes, suggesting that this is a common intrinsic property for evolving networks. This analysis demonstrated that the irregularity of the neutral network in genotype space is related to the underlying mechanism. Since the shape of a neutral network has been suggested to influence evolutionary innovation it is possible that certain mechanisms are favorable from an evolutionary standpoint [19]. This analysis also revealed that counter-balanced regulatory inputs may often be responsible for RSE, and that RSE can be bypassed by means of permissive mutations which buffer the changes that occur in the direct route between two GRN topologies (Figure 3b).

### Validity of the Model

A key assumption for the validity of the model used in this study is that the core networks described do actually control variation in phenotype in natural systems. Intriguingly both the classical and the mutual inhibition type networks can be found as sub-networks of the *Drosophila* gap gene network [17]. The gap gene network of the *Drosophila* blastoderm has been shown to reduce variation in gene expression from Bicoid to the downstream GAP genes by the process of canalization [23], [24]. Indeed knock out of Kruppel, Knirps or Tailless increases this variation [25], [26]. Hence it can be demonstrated that for at least 2 core GRNs evaluated in this study there exists evidence that these networks control variation in gene expression and the resulting phenotype since the Gap genes control the segmentation of the embryo. The model is thus valid to explore evolutionary trajectories in developmental GRNs.

One criticism of the model we have used is that it does not utilize a typical fitness measure. Typical fitness measures involve some aspect of the phenotype that can be considered a selectable trait. For example the sharpness of the stripe of gene expression would be one such measure that could potentially give a selective advantage. These features have important influences on the likelihood that different routes are taken in genotype space. However the most important contribution to fitness is whether the genotype can achieve the function or not irrespective of how “well” it performs the function. Our average functional neutrality is based on the likelihood of a genotype that corresponds to that topology being functional. We have shown average functional neutrality of topologies correlates with the fraction of functional paths through them (Figure 3b). As such it is a valid statistical measure of the amount of RSE and restriction in evolutionary paths within a genotype-phenotype structure.

Furthermore this idea can be illustrated when we envisage our sampling in topology space as a density map of functional genotypes (topology with specific parameters) in underlying continuous genotype space (Figure 3e). Here the regions of space corresponding to the topologies with high average functional neutrality are dense, while those corresponding to the topologies with low average functional neutrality are sparse. For one to travel from a genotype in one dense region to a genotype in the other dense region directly through topologies 2 and 3, statistically many of the routes will involve a non functional intermediate as illustrated by the 2 RSE geometries in figure 3e (this point is confirmed by our mutational walks through parameter space; values on the edges in figure 3b). Hence though we have only scored this topological geometry as a single RSE, in the underlying continuous genotype space it may represent multiple RSEs. In the same way, though we have scored just a single extra dimensional bypass from topology 1 through 5, 6 and 7 to 4, in underlying continuous genotype space there maybe multiple routes as illustrated by the bold arrows in figure 3e. Taken together our measures of RSE and bypass frequency are likely to be underestimates of the actual amounts of RSE and bypasses in these genotype-phenotype maps. This further strengthens our conclusions that RSE and bypasses are frequent in genotype-phenotype maps for higher levels of biological function.

### A General Theory of Permissive Mutations

Our analysis suggests one general theory for permissive mutations in gene regulatory networks and possibly other genotype-phenotype systems. There is an order by which mutations with opposing affect on functionality must occur. A mutation with a minor affect (the permissive mutation) must occur before a mutation with an opposite larger effect to maintain the system in the dynamic range as defined by the system configuration. For example in this work one aspect of the system configuration is the maximum and the minimum of the morphogen gradient which defines a dynamic range between 1 and 0.1. The minor permissive mutation shifts the functionality (stripe of gene expression) to the edge of the configuration limits (edge of the morphogen gradient or spatial boundary in our case). The probability of maintaining function with this mutation is greater than the probability of maintaining function with the large opposing mutation since the large mutation is likely to shift the functionality out of the dynamic range of the system (shift the stripe off the end of the spatial boundary so that the stripe only occurs between two morphogen thresholds both above 1– Figure 3c second panel). However once the permissive mutation has occurred, the scope of the dynamic range of the system is now much larger for the second larger mutation since its affect on functionality (the stripe) will push the system to the opposite side of the configuration limits. Hence the larger mutation can be a viable mutation, but only if the permissive mutation has already occurred. Note such situations would not arise if the morphogen gradient would not have configuration limits and could range from infinity to 0. However such boundaries or limits to the system like the concept of saturation for example are physically unavoidable aspects of GRN systems. As such the concept of smaller permissive mutations followed by larger mutations with opposing effect are probably a common explanation for extra-dimensional bypasses in both genotype-phenotype maps of GRNs and other genotype-phenotype systems.

### Testable Hypothesis

How then can we experimentally test out hypothesis that mutually balanced interactions are responsible for RSE and interactions of opposing action yet differing strength are responsible for permissive mutations? Testing such hypothesis will require going beyond the identification of essential genes (for which data is currently available in multiple species) to a situation where essential individual gene-gene interactions are identified. A complementary experiment for validating our hypothesis would involve constructing our GRNs synthetically. If one could synthetically construct such networks then one could directly explore the affects of adding complementary positive and negative interactions of differing strength. We would predict for example that adding either alone (of similar strength) would diminish function, but in combination should have minimal effect. We would also predict that in general mutations of opposing effect but of different strength maintain function only if added in a particular order (minor permissive mutation first). At least 3 of our core networks have already been built in multiple species and others are currently being constructed [27]–[30]. The power of such constructions is that they only contain the basic core interactions needed for the function rather than other natural networks that have become baroque in nature (contain a more complicated architecture than seems necessary for the function [31]) probably through drift. Indeed our observation that extra dimensional bypasses are common in genotype-phenotype maps for GRNs suggests one explanation for why GRN networks have a baroque structure in the first place since there are many possibilities for adding functionally neutral gene-gene interactions which seemingly are not important for core function.

### Replaying the Tape of Life at Higher Levels of Biological Organization?

How can the results of this study be compared to those results exploring similar phenomena in single proteins? A related phenomenon to permissive interactions with restricted evolutionary paths due to RSE has also been observed for specific examples in protein sequence-function relationships [14]. The existence of such a phenomenon in these diverse contexts suggests that permissive routes through higher genotypic dimensions are a general feature of the evolution of biological systems. However, one of the key findings of studies of evolutionary constraints in single proteins was that many intramolecular combinations are non-viable and therefore the trajectories open to evolution are limited and often the “tape of life” can be replayed such that that evolution often takes the same mutational path [13], [32], [33]. Here we have studied a higher level of biological organization and instead find that there are many more routes between functional genotypes (almost all incidences of 2 topologies linked via RSE have at least one bypass for example). This discrepancy between these conclusions could simply result from the fact that we have used a binary fitness function. Although many routes produce a functional stripe of gene expression, some routes may result in the production of a “better” stripe than others such that those routes are more likely. If a less abstract fitness function was used then involving some aspect of the quality of the stripe this may biased the likelihood of some specific paths over others. Alternatively the discrepancy may result from the fact that when considering a single protein the ways to increase fitness are greatly limited due to pleiotropy and conformational epistasis. Protein sequence space is discrete and any change can have a great effect on multiple aspects of the protein potentially destroying the function. Genotype space by contrast is continuous meaning that changes can be finer allowing for more oblique traverses without loss of function. The tape of life for the evolution of development at the level of GRNs then, may not be as predictable as that for protein function.

## Methods

### Enumerating All GRN Topologies

A topology can be represented in the form of a matrix *w_ij_* where *i* and *j* represent the position in those matrices and values 1, −1 and 0 represent activation, repression and no interaction respectively. We generated all possible matrices that correspond to unlabelled topologies and then removed isometric equivalents by comparing them in all possible permutations. There are 19,683 gene network matrices before non-isometric topologies have been removed and this is reduced to 3,284 topologies in the fully enumerated set.

The morphogen gene is a gene that activates one of the genes of the GRN but is not affected by the GRN. Each GRN topology is represented multiple times with the morphogen feeding into the different genes (exact number depends on the amount of symmetry in the GRN topology). The morphogen is taken account of in the topology generation by extending the GRN matrix (*i = i+1*) to include the input from the morphogen (which is permutated independently). When the morphogen is included the number of isometric topologies increases from 3,284 to 9,710.

### Creating an Atlas of GRNs by Including Explicit Neighbour Definitions

Two GRN topologies are considered neighbours in the atlas if the two GRN topologies are one Hamming distance apart (a single gene-gene interaction change). The Hamming distance can be measured by the following equation where
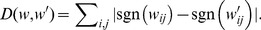
(1)
*D* is the Hamming distance between the matrices of two GRN topologies *w* and *w′* whilst *i* and *j* represent the position in those matrices. The matrices are compared in every permutation and the lowest *D* of those permutations is taken as the Hamming distance. Hence two GRN topologies are neighbours if the gain or removal of any one interaction can transform one of the GRN topologies into the other.

### The Gene Regulation Model

We employed a biologically-verified model of gene regulation for this problem, and therefore adapted the continuous mathematical model developed over the last 20 years by Reinitz et al [34] which quantitatively captures the spatio-temporal dynamics of gap gene patterning in response to the Bicoid morphogen gradient during *Drosophila* embryogenesis. The model is described by
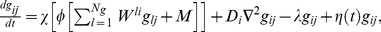
(2)where *g_ij_* is the concentration of the *ith* gene in the *jth* cell, 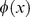 is a function defining the interaction amongst genes (which can take the form of a Michaelis-Menten, sigmoid or other non-linear input function), 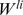 is a matrix containing the strength of gene-to-gene regulation parameters, *M* is the morphogen input described in more detail in the section “configuration of the spatial domain” below, 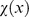 is the Heaviside function (to prevent negative gene product production rates), 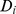 is the diffusion constant for the *ith* gene which we use to represent local cell-cell signaling, 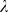 is the decay rate (set to 0.05), and 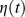 is a noise term which adds uniformly-distributed fluctuations (+/−1%) to the concentration of every gene in every cell at every time step. There is zero auto-correlation in the noise term. The parameters that could vary in the model were regulation 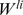, and diffusion 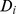. The input function describes the relationship between the activation and inhibition of a gene and its actual expression. The input function used in this work took the form of a Michaelis-Menten function which is defined by
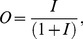
(3)where 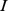 is the total input into the gene and 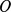 is the output of the function.

### The Discretized Form of the Equations

How the concentration *x* of a gene *i* will change in any given cell *j* at time *t* is described by
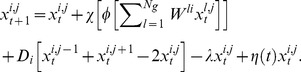
(4)Here 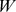 is the interaction matrix described earlier and 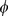 is the input function. 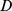 is the diffusion coefficient for each gene 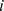 and 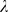 is the decay parameter which is the same for every gene. The noise term 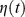 selects a random number within a given range.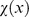 is the Heaviside function where 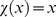 for 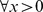 and 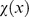 otherwise. Its purpose is to make sure the regulation term can only take positive values.

### Parameter Range Distributions

For each GRN topology 30,000 different parameter sets were tested (a GRN topology with a specific set of parameters we called a genotype). There are up to 12 variable parameters for a 3 gene network; diffusion for each individual gene and then the strengths of the interaction values between the genes. The parameters are chosen randomly though biased towards lower numbers through a logarithmic probability distribution. The logarithmic probability distribution was implemented in order to take account of the fact that a small change in a small parameter value will have a greater effect on a network’s behaviour than a small change in a larger parameter value. The logarithmic probability distribution is described by
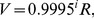
(5)Where 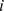 is a random number between 0 and 10,000 and 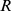 is the parameter range and 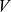 is the resulting parameter value. Parameter ranges are as follows; regulation 0–10 and diffusion 0–0.05.

### Configuration of the Spatial Domain

The simulations take place on a theoretical one dimensional row of 32 cells. Zero-flux boundary conditions are used throughout this work. The simulation starts with every gene in every cell set to have a concentration of 0.1. This was necessary because the noise term used is a percentage noise term and thus if the concentration were always 0 at the start of the simulation then the products of any genes with positive feedbacks without any other input would remain at 0. The simulation is also initiated by the positive input from the morphogen gradient that does not change throughout the simulation.

The morphogen strength was chosen to give an approximate input range to the receiving gene of 10–50% of the maximal activation. The morphogen input is defined by
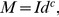
(6)Where 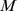 is the morphogen input, 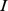 is the morphogen concentration in the left-most cell of the field, 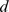 is the reduction of morphogen concentration in each subsequent cell of the morphogen gradient and 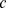 is the cell position. For the 10–50% input range, 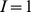 and 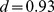 was used for the Michaelis-Menten function.

### Stripe Forming Functional Definition

For a genotype (GRN topology with a specific parameter set) to be considered functional it had to reach an equilibrium (described in Supplementary Methods) and it had to produce a stripe of gene expression for at least one of the genes. For each gene we measured an abstraction of its gene expression over the one-dimensional field where each cell was defined as low or high. We defined a cell as low if the gene expression was below 10% of the maximum possible allowed by the model. We defined a cell as high if the gene expression was above 10% of the maximum gene expression allowed by the model. A gene was considered to have a stripe pattern if it had a single region of low for 2 consecutive cells followed by a single region of high for a maximum of 16 consecutive cells followed by a single region of low for at least 2 consecutive cells. The two low regions must occur at the extremities of the field. The definition is intentionally loose in the sense that the single stripe can be of any width up to 16 cells and be in any position in the spatial domain. This is because we are interested in the basic design principles of the system, not the details of how to control a specific width. Functional parameter sets that can produce the single stripe of gene expression we term “solutions”. Hence a single topology has multiple genotypes and can have multiple solutions. The number of solutions that each topology has is a measure of its mutational robustness. A GRN topology must have at least one solution to be considered functional.

### Splitting the Functional Topologies into Subsets each Responsible for a Different Mechanism and Creating Average Functional Neutrality Landscapes

In order to explore whether changes in the mechanism by which a GRN is functioning is responsible for the prevalence of RSE in an average functional neutrality landscape, we split the functional GRN topologies into 6 categories, each corresponding to the 6 different mechanisms we identified in our previous study [17]. If a GRN topology could perform a mechanism for at least one parameter set then it was included in that particular subset. Therefore a GRN topology can be present in multiple subsets. GRN topologies were assigned to mechanistic classes using the method for mapping mechanisms to the complexity atlas described in Supplementary Methods. Each subset is then used to build an average functional neutrality landscape for each particular mechanism. The total number of functional GRN topologies was 471. The number of GRN topologies in each of the average functional neutrality landscapes was as follows; Incoherent Feed-Forward type 1 (97), Mutual Inhibition (44), Frozen Oscillator (27), Overlapping Domains (56), Bi-stable (109) and Classical (39). Topologies are assigned an average functional neutrality score based on their parameter robustness (Number of parameter sets that successfully produced the stripe of gene expression). Topologies are vertices in the landscapes and they are connected by edges based on the neighbor definitions of the atlas described earlier.

### Calculating the Extent of RSE

In order to calculate the extent of RSE in the individual average functional neutrality landscapes we analyzed all pair-wise combinations of topologies in the average functional neutrality landscape to see if they conform to the V-shaped viability/fitness geometry. The V-shaped viability/fitness geometry of RSE is defined as two GRN topologies (topologies A and B) that are two hamming distances apart that have exactly two direct intermediate topologies. These intermediate topologies both must have significantly lower average functional neutrality than topologies A and B. Significantly low average functional neutrality is defined by
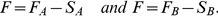
(7)Where 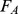 is the average functional neutrality of topology A (which is defined as the number of functional parameter sets for that topology) and 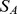 is the standard deviation of the average functional neutrality of topology A, assuming a binomial distribution. The standard deviation of a binomial distribution is described by
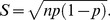
(8)Where 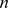 is the number of trials and p is the probability of functionality (the true average functional neutrality). Since we do not know the true average functional neutrality, this measure is bootstrapped using the measured average functional neutrality 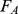 as a proportion of the total number of parameter sets tested (number of trials, 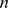) giving
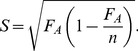
(9)


### Calculating the Extent of Permissive Mutations/extra-dimensional Bypasses

In order to calculate the extent of extra-dimensional bypasses between pairs of GRN topologies that have the RSE V-shaped viability/fitness geometry we analyzed all mutational routes between these pairs of topologies. For a route to be considered an extra-dimensional bypass every GRN topology within the route must have average functional neutrality within one standard deviation of topologies A and B as described in equation 7. For each pair-wise combination of GRN topology showing the RSE geometry we record the length of each extra-dimensional bypass (measured by the number of gene-gene interaction changes). The direct permissive mutations are those with a bypass length of 4 since they involve the 2 original mutations required to change topology A into B (and vice versa) and the two other mutations that involve the addition and removal of the permissive mutation.

### Performing Mutational Walks between Topologies

We measured the fraction of mutations from a functional parameter set of one topology to a functional parameter set of another topology (specifically in the direction of the arrows in figure 3b). We start with a functional parameter set of our reference topology and remove or add the appropriate interaction. If we add an interaction, we give the new interaction the appropriate sign and with a random value using the criteria that we describe in parameter range distributions above. If we remove an interaction the value is simply set to 0. We then re-simulate this mutated genotype and ask if it is functional using our functional stripe definition described above. We do this 100 times for every specific parameter set and calculate the proportion of tests that are functional. For example we perform 74,400 tests of mutating topology 1 to topology 5. The proportions of functional tests are found in brackets on the edges of figure 3b.

## Supporting Information

Figure S1 **Summarizing the key results of the previous study** [**17**] **.** A complexity landscape identifies core mechanisms responsible for generating a stripe of gene expression. Vertices are topologies and edges connect topologies one hamming distance apart (one gene-gene interaction change). Topologies are spaced manually in the x-axis to reduce edge crossing and in the y-axis by their complexity (number of gene-gene interactions). Stalactites of complexity emerge out of the bottom of the landscape converging to minimal core topologies that represent distinct mechanisms. Mechanisms were mapped to the complexity landscape by coloring topologies according to their mechanism class (See Supplementary Methods). Topologies were colored Light green (A: Incoherent feed-forward type 1 mechanism), Light Blue (B: Mutual inhibition mechanism), light red (C: Frozen oscillator mechanism), Dark green (D: Overlapping domains mechanism), Dark blue (E: Bi-stable mechanism) or Dark red (F: Classical mechanism). Topologies were colored yellow if they were capable of acting via multiple mechanisms depending upon their exact parameter set. The corresponding core topologies of these mechanisms (those at the very bottom of the stalactite) are shown below the complexity landscape. 3 examples space-time behaviors of each mechanism are also shown. Here the spatial dimensions represent time and space and the intensity of red, green or blue based on the expression value of that gene at that time in that cell. The final gene expression graphs at equilibrium are shown below (corresponding to the bottom space time plot).
(TIF)

Figure S2 **Topology space is a discrete representation of an underlying continuous genotype (parameter) space.** The concept of a topology space (adapted from reference 17). ***(a)*** A GRN topology where two of the gene-gene interactions α and β correspond to the parameter space in *(b)*. ***(b)*** A parameter space of the two parameters α and β. Dots are random parameter sets from this space. ***(c)*** A topology space is created if all values of α and β that are positive are considered gene-gene activations, those values of α and β that are negative are considered gene-gene repressions and those values of α and β that are 0 are considered to generate no gene-gene interaction. Regions of parameter space corresponding to the different topologies are indicated by the different colored circles surrounding the topologies and the different colored dots in *(d)*. Where topologies differ by a single gene-gene interaction (one Hamming distance) they are linked by a blue line. Such links connect regions of close parameter space.
(TIF)

Figure S3 **Not all topology changes represent viable steps in an evolutionary walk on a neutral network at the level of the underlying genotype space.** Two examples are shown. Boxes represent a simplified version of the parameter spaces of 3 topologies called A, B and C. Black circles represent the functional parameter volumes. Thick arrows represent viable mutational steps and dashed arrows represent unviable steps. (Top) The two topologies (A and B) each work by a single but different mechanism. Changing A into B or vice versa then leads to a non-viable region of parameter space. (Bottom) The topology (B) can generate the gene expression pattern with two mechanisms. Two other neighboring topologies (A and C) can function but using either one of these two mechanisms. Changing from either A or C to B is viable. However changing from A to B to C or vice versa is only possible if the change between the viable regions of topology B involves a change in only a single parameter.
(TIF)

File S1 **Consisting of Supplementary Note and Supplementary Methods.**
(DOCX)
