# Supplementary figures and images for: Mechanistic Explanations for Restricted Evolutionary Paths That Emerge from Gene Regulatory Networks
Source: PLoS One. 2013 Apr 17;8(4):e61178. doi: 10.1371/journal.pone.0061178 (PMC3629181; doi:10.1371/journal.pone.0061178)

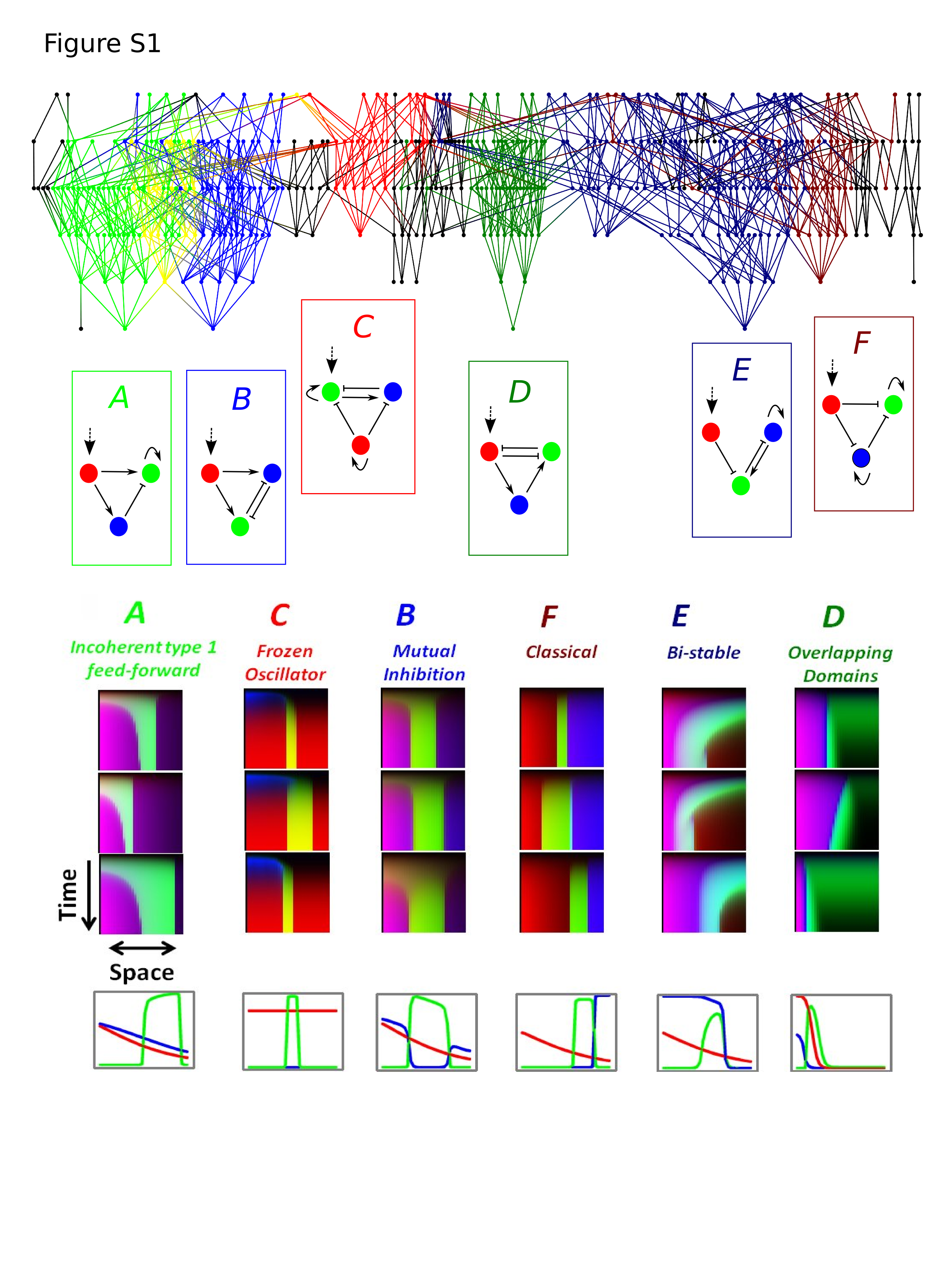

Supplement: Figure S1 — Summarizing the key results of the previous study [17] . A complexity landscape identifies core mechanisms responsible for generating a stripe of gene expression. Vertices are topologies and edges connect topologies one hamming distance apart (one gene-gene interaction change). Topologies are spaced manually in the x-axis to reduce edge crossing and in the y-axis by their complexity (number of gene-gene interactions). Stalactites of complexity emerge out of the bottom of the landscape converging to minimal core topologies that represent distinct mechanisms. Mechanisms were mapped to the complexity landscape by coloring topologies according to their mechanism class (See Supplementary Methods). Topologies were colored Light green (A: Incoherent feed-forward type 1 mechanism), Light Blue (B: Mutual inhibition mechanism), light red (C: Frozen oscillator mechanism), Dark green (D: Overlapping domains mechanism), Dark blue (E: Bi-stable mechanism) or Dark red (F: Classical mechanism). Topologies were colored yellow if they were capable of acting via multiple mechanisms depending upon their exact parameter set. The corresponding core topologies of these mechanisms (those at the very bottom of the stalactite) are shown below the complexity landscape. 3 examples space-time behaviors of each mechanism are also shown. Here the spatial dimensions represent time and space and the intensity of red, green or blue based on the expression value of that gene at that time in that cell. The final gene expression graphs at equilibrium are shown below (corresponding to the bottom space time plot). (TIF) [file pone.0061178.s001.tif]

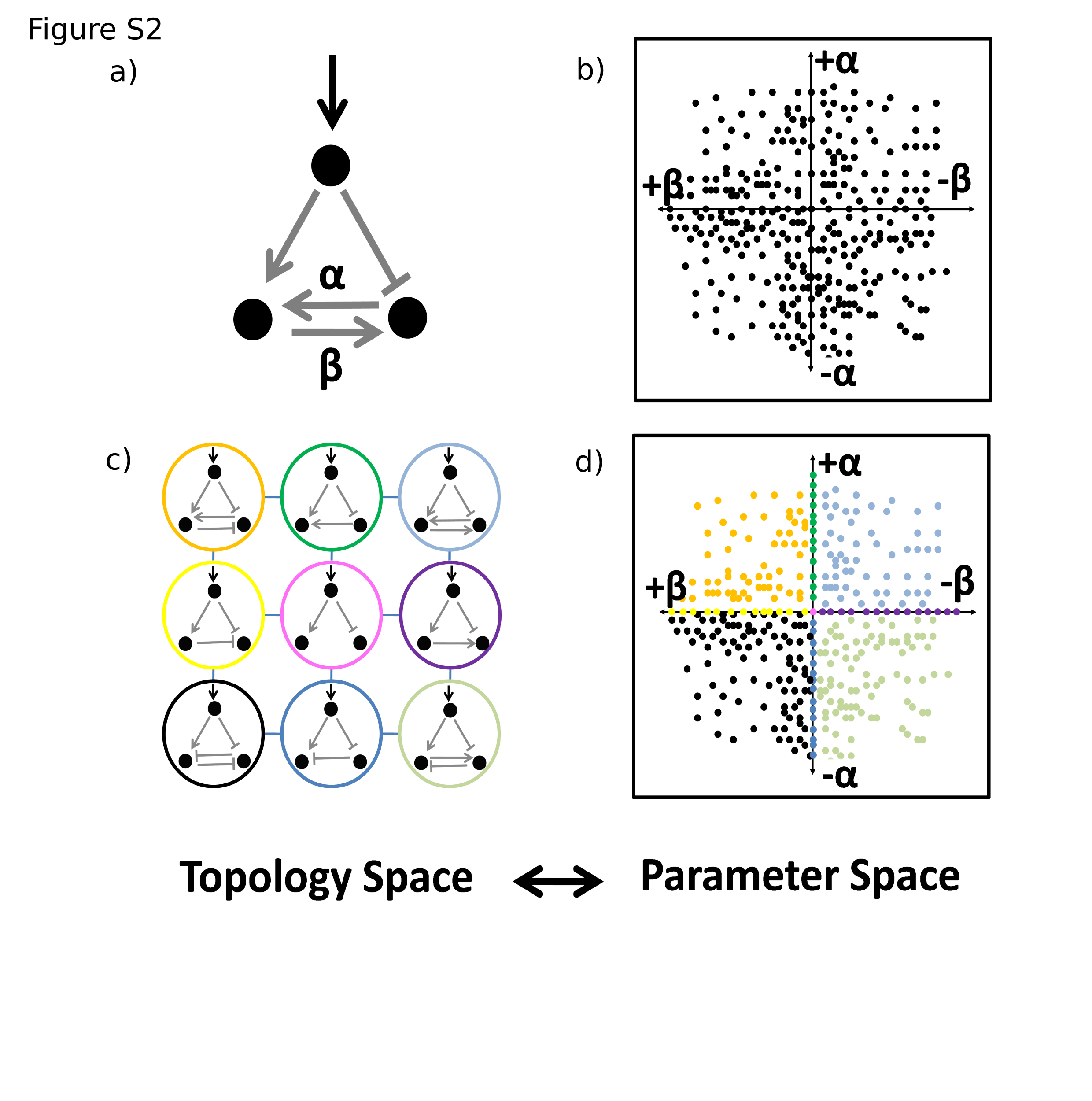

Supplement: Figure S2 — Topology space is a discrete representation of an underlying continuous genotype (parameter) space. The concept of a topology space (adapted from reference 17). (a) A GRN topology where two of the gene-gene interactions α and β correspond to the parameter space in (b). (b) A parameter space of the two parameters α and β. Dots are random parameter sets from this space. (c) A topology space is created if all values of α and β that are positive are considered gene-gene activations, those values of α and β that are negative are considered gene-gene repressions and those values of α and β that are 0 are considered to generate no gene-gene interaction. Regions of parameter space corresponding to the different topologies are indicated by the different colored circles surrounding the topologies and the different colored dots in (d). Where topologies differ by a single gene-gene interaction (one Hamming distance) they are linked by a blue line. Such links connect regions of close parameter space. (TIF) [file pone.0061178.s002.tif]
