# Supplementary material for: Mechanistic Explanations for Restricted Evolutionary Paths That Emerge from Gene Regulatory Networks
Source: PLoS One. 2013 Apr 17;8(4):e61178. doi: 10.1371/journal.pone.0061178 (PMC3629181; doi:10.1371/journal.pone.0061178)
Supplement: File S1 — Consisting of Supplementary Note and Supplementary Methods. (DOCX) [file pone.0061178.s004.docx]

**Mechanistic explanations for restricted evolutionary paths that emerge from gene regulatory networks**

**Supporting Information**

**James Cotterell and James Sharpe**

**Supplementary Note**

**Supplementary Methods**

**Supplementary Note:**

Description of the previous study

Attempting evolutionary walks through the indirect route.

**Supplementary Methods:**

1. Criteria to assess whether a simulation produces a gene expression pattern and is robust with respect to developmental noise.
2. Splitting functional topologies into mechanism groups.

**Supplementary Note**

**Description of the previous study**

In our previous study (Cotterell and Sharpe, 2010) we explored the full complement of GRN’s mechanisms that could convert a continuous graded morphogen input into discrete spatial domains. In particular we asked if the GRN topologies were capable of generating a stripe of gene expression, a common pattern observed during development.

*Assessment of Topology functionality (Full details in methods):*

We simulated all possible topologies in a one dimensional field of 32 cells, with zero-flux boundary conditions. 30,000 parameter sets were tested for each GRN topology and the output assessed to see if that topology was functional. Variable parameter sets included strength of gene-gene regulation and diffusion of gene products (ranges 0-10 and 0-0.05 respectively). Fixed parameters included the rate of decay of gene products (0.05). Parameter selection was biased towards smaller values by means of a logarithmic function. A Michaelis-Menten input function with the sum and filter approach was used to model the input-output relationship of genes. A small noise term was added that changed the gene expression value for each gene in each cell at every time point of the simulation. Topologies with specific parameter sets were simulated until they reached equilibrium or until 1000 iterations had passed (whichever was shortest). For a topology to be considered function it has to produce a noise robust stripe of gene expression that had reached equilibrium for at least one set of parameters tested. The details of how a topology with a specific parameter set was judged to be robust with respect to noise and at equilibrium are described in supplementary methods.

*Generating a complexity landscape to identify mechanisms:*

We identified mechanisms by identifying the minimal topologies capable of performing the stripe function. This was achieved by generating a complexity landscape to identify core topologies responsible for different mechanisms (Figure S1). These core topologies were found to be working in distinct ways, confirming the complexity landscapes ability to elucidate different mechanisms. We demonstrated that there were at least 6 different mechanisms for generating a single stripe of gene expression in a one dimensional row of cells and that these mechanisms map to discrete regions of genotype space.

*How the mechanisms work (From reference 17):*

***A*: Incoherent type 1 feed-forward**

**Description:** (Stage 1): The red gene is activated by the morphogen and hence starts to mimic its gradient pattern. The red gene activates the green gene, and switches on the green genes positive feedback above a certain threshold. (Stage 2) A combination of time and dose leads to the concentration of the repressing blue gene product building up to a high enough concentration for it to force the green gene product down, but only on the left hand side where the concentration is higher. (Stage 3) In the central region, the repression from the blue gene product is lower allowing the green gene product to reach a high steady state.

***B*: Mutual Inhibition**

**Description:** One of the real mechanisms found to be involved in *Drosophila* anterior-posterior patterning (Stage 1). The red gene product mimics the expression pattern of the morphogen. The green gene is activated more strongly than the blue gene and hence switches on earlier on the anterior side where the Red activating gene product is higher. (Stage 2) Eventually the blue gene product builds up to a high enough concentration to start forcing the green gene product down on the very anterior side where the concentration is highest. (Stage 3) The result is a final gene expression pattern where the blue and green gene products form two mutually exclusive expression zones.

***C*: Frozen Oscillator**

**Description:** (Stage 1): The morphogen sets the green gene and the blue gene products oscillating because they are in a negative feedback loop. The phase of the oscillation is different in different cells because of the difference in the strength of the morphogen activation. (Stage 2): The red gene product starts to build up everywhere in a uniform distribution because of its positive feedback. It represses both the green and blue gene and stops the oscillations forcing both genes off, except in the central region which are in a phase of the oscillation allowing the concentrations to reach a high steady state.

***D*: Overlapping Domains**

**Description:** This mechanism is completely dependent upon diffusion. The stripe gene is activated and inhibited by the two genes. Due to the activator having a higher diffusion constant than the inhibitor, the expression domain of the activator extends further than that of the inhibitor allowing a region for a stripe to form. (Stage 1) The red gene product starts to form a gradient as it is activated by the morphogen. The red gene strongly activates the blue gene giving it a similar expression profile though at a higher concentration. The blue gene product activates the green gene and the red gene inhibits the green gene meaning the green gene product can only start to increase in concentration on the right hand side. The green gene inhibits the red gene which causes the sharp threshold break in the red gradient. (Stage 2) The green gene forces the red gene completely off on the right hand side, in turn leading to decay of the blue gene product and then the green gene product. (Stage 3) Only in the overlap region where the blue gene is activating the green gene and where there is no repression from the red gene can a stripe form.

***E*: Bi-stable**

**Description:** (Stage 1) The red gene is activated by the morphogen and thus its product also forms a gradient. The blue gene activates itself and thus starts to switch on everywhere. The blue gene also activates the green gene whose product will thus start to increase in concentration, but only on the right hand side as it is repressed by the red gene on the left hand side. (Stage 2) The green gene product can build up to a high enough concentration on the right hand side to start to force down the blue gene product. (Stage 3)The green gene however is also dependent upon the blue gene for activation and thus after a delay its product concentration also starts to fall. The result is a single stripe of gene expression.

***F*: Classical**

**Description:** One of the real mechanisms found to be involved in *Drosophila* anterior-posterior patterning (Stage 1). The red gene is activated by the morphogen and thus it’s product also forms a gradient. (Stage 2) The blue and the green genes activate themselves and they start to switch on where the repression from the red gene is lowest. (Stage 3) The blue gene also represses the green gene meaning it is forced off at the very right. Only in the central zone where the repression from the red and the blue gene is lowest can the green gene product reach a high steady state.

**Attempting evolutionary walks through the indirect route:**

Simply showing that there exists a functional parameter volume for all topologies on a route between topology 1 and 4 does not mean that there exists a functional evolutionary trajectory from 1 to 4 since those topologies on route could be using very different regions of parameter space. If they are using different parameter volumes then a single mutational change may not move from one functional parameter domain to another. An illustration of this idea is shown in figure S3.

To test whether there really was a functional route between topologies 1 and 4 we performed evolutionary walks from functional parameter sets of Topology 1 to functional parameter sets of topology 4 (Figure 3a and b). We focused on the regulation parameters. Specifically we attempted walks from every viable parameter set of GRN topology 1 in figure 3 (744 parameter sets). For each viable parameter sets we attempted 100 walks (74,400 walks attempted in total). At each step of the walk, we converted the topology to the next topology in the path (1 to 5 to 6 to 7 to 4) by removing or adding an interaction. If an interaction was added, its value was randomly chosen within the normal range of the model (See Methods). For each newly generated topology we simulated it to test whether it could generate the single stripe of gene expression. If it could, then the walk was continued, if not then the walk failed. Only walks that reached the final topology (4) were considered functional. The number of functional steps were as follows; 1>5 (229), 5>6 (17), 6>7 (4) and 7>4 (2).

**Supplementary Methods**

**1. Criteria to assess whether a simulation produces a gene expression pattern and is robust with respect to developmental noise.**

In this work we are interested in GRN topologies that are capable of generating a stripe of gene expression. However, because our laboratory is interested in many types gene expression pattern other than singles stripes, we first apply filters that assess whether the result of a simulation produces a gene expression pattern at all (i.e. non-uniform over the field of cells) and whether it is robust to developmental noise. Once these filters have been applied we assess whether the resulting noise robust gene expression pattern is indeed a stripe. Only simulations that result in a single stripe of gene expression are considered in this work. If it does not have a stripe pattern then it may be considered for other projects.

*The pattern filter:*

A patterning score describes how much ‘pattern’ or spatial heterogeneity there is. A patterning score is calculated for each gene of the GRN topology. It is measured as the Euclidean distance from the mean concentration of each gene over the field of cells to the actual concentration of the gene product in each cell using

$P=\frac{1}{N}\sum_{i=1}^{N} \sqrt{\left( V_{i}-\bar{V} \right)^{2}}$. (10)

Here *P* is the patterning score, *V* is the vector of the gene concentration over the field of cells, $\bar{V}$ is the average value of the vector *V* and *N* is the number of cells. The score is thus normalized to the number of cells.

*The noise filter:*

The fragility score is an inverse measure of the noise robustness of the gene expression time-course. The fragility score is also calculated for each gene of the GRN topology. Each genotype is simulated multiple times (always 4 in this project). Each individual simulation, we term a noise run. An average noise run is generated from these four noise runs. The fragility score is measured as the total Euclidean distance between each of the noise runs and the average noise run using

$F=\frac{1}{\left( NR \right)}\sum_{i=1}^{N} \sum_{j=1}^{R} \sqrt{\left( A_{i,j}-\bar{A}_{i} \right)^{2}}.$ (11)

Here *F* is the fragility score, *A* is the array of gene product concentrations of each cell *i* in each noise run *j.* $\bar{A}_{i}$ is the average value for cell *i* between the noise runs. *N* is the number of cells and *R* the number of noise runs. The score is normalized to the number of cells and the number of noise runs.

We close the following thresholds as suitable for selecting a gene expression time-course that was considered patterned and robust with respect to noise:

- The pattern score must be above 10. Below this threshold there are only weak patterns.
- The pattern score divided by the fragility score must be above 10. We observed a general correlation between the pattern and fragility score. This was expected since noise is also read as a heterogeneity and thus pattern. Therefore, we chose a conservative ratio of 10 as the threshold defining a patterned and robust gene expression time-course.

*The equilibrium filter:*

In this work we restricted our analysis to gene expression time-courses that reached equilibrium. It should be noted that there is no reason to assume that equilibrium gene expression time-courses are any more biologically relevant than those that do not reach equilibrium. To test whether the simulation had reached equilibrium, another score measures the Euclidean distance between the gene expression pattern at two time points with 50 iterations of the simulation between them. This measure is made every 50 time steps of the simulation. This score was normalized to the number of genes and cells in the model. A difference threshold of 2 was chosen for the dynamic to be defined as in equilibrium and the simulation stopped. A maximum of 1,000 time steps are performed. The criterion is given by the condition that

$2>\frac{1}{\left( GN \right)}\sum_{i=1}^{G} \sum_{j=1}^{N} \sqrt{\left( X_{i,j,t}-{Xi,j,}_{t-50} \right)^{2}},$ (12)

where *X* is the array of gene product concentrations for each gene *i* in each cell *j* for each time point *t.* *G* is the number of genes and *N* is the number of cells. The score is normalized to the number of genes and cells.

**2. Splitting functional topologies into mechanism groups.**

Functional topologies could be included in multiple mechanism groups. If a functional topology only contains the core topology of only one of the 6 basic mechanisms (A-F in figure S1), then that topology with all of its functional parameter sets is placed in that mechanism class. If a topology contains the core topologies of multiple basic mechanisms, then the space-time behavior of each parameter set of that topology is analyzed to split the parameter sets into the different mechanism classes. The space-time behavior is compared to all space time behaviors in the standard set and the parameter set is added to the class with the most similar space time behavior.

First, to generate the standard space-time behavior set for each class, all functional parameter sets for the 6 core topologies are simulated (A-F in figure S1; until equilibrium). These space-time gene expression values for all parameter sets of each core topology constitute the standard set. To analyze which mechanism a particular parameter set of a topology belongs to it is first simulated and at the final time point (at equilibrium) the cells in the center of the 3 regions low-high-low are calculated (using the definition in the section ‘stripe forming definition’ in the main methods section). The gene expression over time of all 3 genes of these 3 reference cells are then compared to the corresponding cells of every standard space-time behavior (only the 3 reference cells are used for this analysis as it is computationally intensive). The Euclidian distance is measured and the parameter set is assigned to the mechanism category that has the most similar behavior (minimum Euclidean distance).Hence if for example a topology had 800 parameter sets but contained both the core topologies of 2 different mechanisms, then that topology would be assigned to both mechanisms but with 200 parameter sets assigned to one mechanism and 600 to the other for example.
